# Modified Quick Change Method for Norepinephrine Syringe Exchange in Critically Ill Patients: A Quasi-randomized, Non-inferiority Trial

**DOI:** 10.7759/cureus.75082

**Published:** 2024-12-04

**Authors:** Reiko Iwamoto, Seiya Kanou, Eiji Nakatani, Yoshiaki Masuda, Rina Sano, Motohiro Asaki, Mika Matsunaga

**Affiliations:** 1 Department of Nursing, Fujieda Municipal General Hospital, Fujieda, JPN; 2 Department of Emergency Medicine and Critical Care Center, Fujieda Municipal General Hospital, Fujieda, JPN; 3 Graduate School of Public Health, Shizuoka Graduate University of Public Health, Shizuoka, JPN; 4 Department of Biostatistics and Health Data Science, Graduate School of Medical Science Nagoya City University, Nagoya, JPN

**Keywords:** critical care, modified quick change method, norepinephrine, syringe exchange, vasopressors

## Abstract

Background: This study aimed to verify that the modified quick change (mQC) method of syringe exchange in critically ill patients receiving a continuous intravenous norepinephrine infusion is not inferior to the conventional double pumping (DP) method.

Methods: This non-blinded, quasi-randomized, non-inferiority trial was conducted in a single hospital from August 1, 2023 to February 29, 2024. Adult patients aged 18 years or older who were admitted to the emergency ward and received a continuous intravenous norepinephrine infusion were eligible for inclusion. This study utilized a convenience sample of all eligible patients admitted during the study period. Patients were allocated to the mQC group or the DP group based on their month of admission. The primary endpoint was defined as the difference between the minimum mean arterial pressure during the 10 minutes after norepinephrine exchange and the mean arterial pressure before exchange. Non-inferiority was established if the lower limit of the 95% confidence interval was within the prespecified margin of 10 mm Hg.

Results: Thirteen patients in the mQC group and eight in the DP group were analyzed. The mean value of the primary endpoint was −8.92 mm Hg in the mQC group and −10.63 mm Hg in the DP group. The 95% confidence interval for the difference between the two groups ranged from 0 mm Hg to 8.50 mm Hg, which was within the prespecified margin. Therefore, the non-inferiority of the mQC method was confirmed.

Conclusion: The mQC method of syringe exchange was not inferior to the DP method in critically ill patients receiving a continuous intravenous norepinephrine infusion. Given its comparable safety profile and simpler procedure, the mQC method has the potential to become a standard exchange technique. However, future large-scale randomized controlled trials are required to verify our findings.

## Introduction

Patients subjected to excessive invasive physiological stress due to various diseases frequently develop hypotension because of vascular smooth muscle relaxation, which leads to decreased tissue perfusion, potentially causing organ dysfunction. To maintain adequate tissue perfusion, administration of both fluids and vasopressors is essential [1,2]. This pathophysiological state is known as distributive shock, which is characteristic of conditions such as sepsis [2,3]. Reliance on vasopressors is particularly high in critically ill patients with shock. Hemodynamic parameters in these patients are extremely sensitive to even slight changes in infusion rates [4].

Norepinephrine is the most widely used vasoactive agent in shock treatment owing to its potency and selective action on vascular alpha (α)-receptors [5]. It is considered the first-line treatment for various types of shock [6,7]. Intravenous administration of norepinephrine requires the use of syringe pumps to maintain a constant flow rate. However, the syringes used are typically small and must be replaced frequently. Given norepinephrine’s short half-life [8-10], syringe changes may cause hemodynamic fluctuations [11]. Therefore, they must be performed cautiously.

Although various methods for norepinephrine syringe exchange have been proposed, clear guidelines on which one to use have not been established. One widely known technique is the double pumping (DP) method [9,12], which uses two syringe pumps. While the first pump with the nearly depleted syringe continues to operate, a second pump with a full syringe is initiated. Norepinephrine is administered simultaneously from both pumps, with gradual flow rate adjustments, until the first pump is eventually stopped. Although this is the most frequently used exchange method in Japan [13], studies suggest that it can cause decreases in mean arterial pressure ranging from 15.4% to 18.2% [14]. Moreover, the procedure is complex, which places a burden on the nursing staff and increases the risk of error [13].

The quick change (QC) method also uses two syringe pumps but involves using a three-way stopcock to switch from the line connected to the nearly empty syringe to the line connected to the full syringe. Unlike the DP method, the QC method does not involve simultaneous catecholamine administration, as the nearly empty syringe is immediately removed after switching the stopcock. The QC method is considered simpler, causes smaller blood pressure fluctuations [14,15], and reduces exchange time.

However, friction between the barrel and plunger of a new syringe may affect infusion rate and concentration [16]. To address this, a new technique has been developed in which 4 mL of norepinephrine is discarded from the newly filled syringe before connecting it to the infusion line [17]. Moving the plunger by 4 mL helps eliminate the friction characteristic of new syringes.

We have named the exchange method that combines the QC method with the 4 mL discard technique as the modified QC (mQC) method and have hypothesized that it is not inferior to the conventional DP method in terms of its impact on blood pressure. This study aimed to test this hypothesis and establish a safer method for norepinephrine syringe exchange.

## Materials and methods

Trial design and ethical and research approvals

This single-center, non-blinded, quasi-randomized, non-inferiority trial was conducted at Fujieda Municipal General Hospital in Fujieda City, Shizuoka Prefecture, Japan. The trial was registered with the University Hospital Medical Information Network (UMIN) Clinical Trials Registry (identifier: UMIN000054663) on August 17, 2023, and approved by the Clinical Ethics Committee of Fujieda Municipal Hospital (approval number, R05-17). All procedures were performed in accordance with the ethical standards of the institutional and national research committees and with the 1964 Helsinki Declaration and its later amendments.

Fujieda Municipal General Hospital is a 564-bed tertiary emergency medical facility located in central Shizuoka Prefecture that services approximately 450,000 people. In Japan, medical facilities designated as emergency hospitals by prefectural governors are classified as primary, secondary, or tertiary emergency centers based on the severity of illness they can accept and treat (mild, moderate, and severe illness, respectively). At our tertiary hospital, severely ill patients requiring emergency care are managed by the emergency department and admitted to the emergency ward.

In the emergency ward, norepinephrine syringes are standardized to 5 mg diluted in 45 mL of normal saline and administered via continuous intravenous infusion using a syringe pump. The indications for norepinephrine use and its administration route are determined at the discretion of the treating physician. Norepinephrine syringe exchanges are performed by emergency ward nurses as needed. As the study was a comparison of norepinephrine exchange techniques, blinding and randomization were not possible. Therefore, quasi-randomization was performed by allocating exchange methods according to the month of admission. A non-inferiority trial design was used because the mQC method is considered simpler and less prone to nursing staff errors than the conventional DP method, which has been our hospital’s standard exchange procedure.

Participants

The study included adult patients aged 18 years or older who were admitted to the emergency ward and received continuous intravenous norepinephrine infusion between August 1, 2023, and February 29, 2024. Those admitted to the emergency ward but managed by departments other than emergency medicine (such as cardiology or neurosurgery) were excluded. Participants were registered after the attending nurse obtained written informed consent from the patient or their family. If participation was declined, norepinephrine exchanges were performed using the method specified by hospital regulations.

Allocation

Patients admitted in September, October, and December 2023 and February 2024 were assigned to the mQC group, while those admitted in November 2023 and January 2024 were assigned to the DP group. As per the ethics committee’s guidance, September and October served as a safety confirmation period for the mQC method, resulting in two consecutive months of intervention group allocation. If more than two mQC method cases did not meet the safety monitoring criteria during this period, the method would have been deemed unsafe.

Data collection

Data were collected only for the first norepinephrine syringe exchange. Non-invasive blood pressure measurements were recorded at one-minute intervals from one minute before the exchange to 10 minutes after. The attending nurse recorded the readings on a designated form. Information regarding sex, age, underlying condition, norepinephrine administration route (central or peripheral venous line), and norepinephrine infusion rate was obtained from the electronic medical record. Patients who declined participation in the trial were treated as excluded cases. Those whose norepinephrine administration ended before the first syringe exchange were treated as dropouts.

Syringe exchange

In the mQC group, 4 mL of norepinephrine from the newly filled syringe was discarded before connecting it to the three-way stopcock attached to the original norepinephrine infusion line. The stopcock was then switched to end the original norepinephrine infusion and start the new one at the same infusion rate.

In the DP group, a new syringe filled with norepinephrine was connected to a separate three-way stopcock on the norepinephrine infusion line. The new infusion was then started at the same rate, resulting in simultaneous administration from two routes. Nurses measured blood pressure every minute, reducing the original norepinephrine infusion by 1 mL/hour when systolic blood pressure increased by 5 mm Hg or more. The original infusion was removed when its flow rate reached 0 mL/hour.

**Figure 1:**
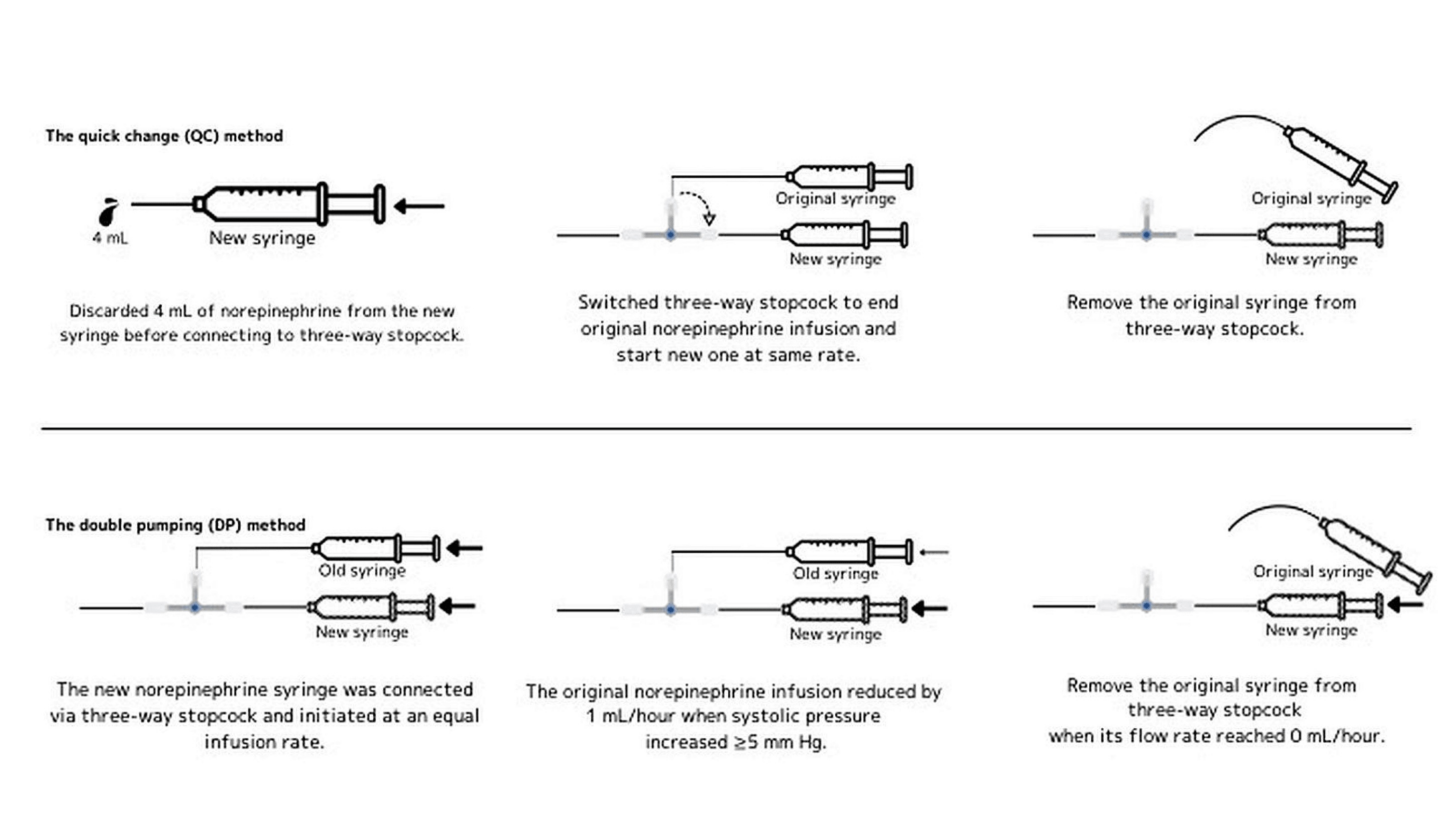
Syringe exchange method Image credits: This is an original illustration created entirely by the authors.

In both groups, the norepinephrine infusion rate was stabilized by maintaining infusion of another fluid at 10 mL/hour through the same infusion route. Syringe pumps were positioned at the same height as the patient’s chest (Figure 1).

Safety monitoring criteria

Predefined safety criteria were monitored by the attending nurse and ward charge nurse. After norepinephrine exchange, the attending physician was notified if systolic blood pressure decreased by 30 mm Hg or more for over two minutes; heart rate increased or decreased by 30 beats/minute or more for over two minutes; the attending nurse deemed additional intervention was necessary; the attending physician would then assess the situation and decide whether to continue the trial.

Outcome

The primary endpoint was defined as the difference between the minimum mean arterial pressure during the 10 minutes after the norepinephrine exchange and the baseline mean arterial pressure before the exchange. No secondary endpoints were established.

Sample size

Based on previous studies [14], we set the difference in mean values of the primary endpoint between the two groups at 0 mm Hg with a non-inferiority margin of 10 mm Hg. The standard deviation was assumed to be 10 mm Hg in both groups. Using a one-sided test (normal approximation) with a type I error rate of 0.05 and power of 0.8, the minimum required sample size was calculated as 13 per group, for a total of 26.

Statistical analysis

Statistical analyses were performed using R software version 4.3.2 (The R Foundation, Vienna, Austria). Non-normally distributed continuous variables were described using the median with interquartile range (IQR), while normally distributed continuous variables were described using the mean with 95% confidence intervals. Categorical variables were described using counts with percentages. T-tests were used for comparing means. The null hypothesis was ∣P1−P0∣ ≥10 mmHg. (where P0 = mean outcome in the intervention group and P1 = mean outcome in the control group). Therefore, if the upper limit of the 95% confidence interval for |P1−P0| did not exceed 10 mm Hg, we rejected the null hypothesis and concluded non-inferiority.

Per-protocol analysis was performed. All patients admitted to the emergency ward and started on continuous intravenous norepinephrine infusion during the specified observation period were allocated to either group. After allocation, those whose norepinephrine administration ended before the first exchange and those whose blood pressure became unmeasurable after the first exchange were excluded from the analysis. No imputation was performed for missing values.

## Results

Study participants

**Figure 2:**
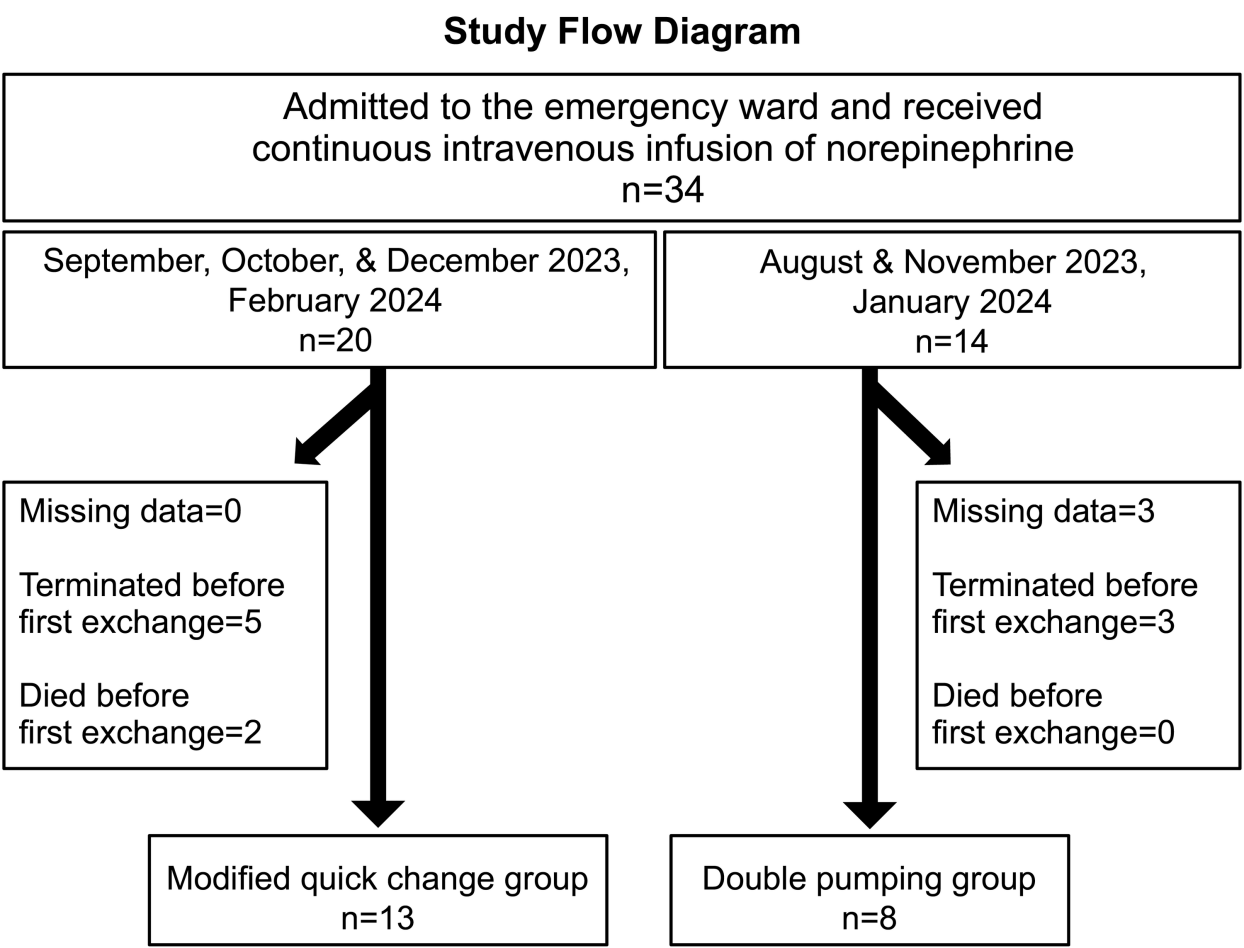
Study flow diagram

Thirty-four patients were enrolled; 20 were allocated to the mQC group and 14 to the DP group based on criteria. Seven mQC patients were excluded from the analysis, five owing to termination before the first exchange and two because they died before the first exchange. Six DP patients were also excluded, three owing to missing data and three because of termination before the first exchange. Finally, 13 mQC group patients and eight DP group patients were included for analysis (Figure 2).

Patient characteristics

The median age of the participants was 73.0 years (IQR, 65.5-78.5) in the mQC group and 80.0 years (IQR, 72.0-85.0) in the DP group. Fourteen participants (66.7%) were men, and the most common underlying condition was infection (eight patients, 38.1%). In the DP group, seven patients (87.5%) were not sedated during infusion. Patient characteristics are presented in Table 1.

**Table 1:** Patient characteristics IQR: interquartile range; mQC: modified quick change; DP: double pumping

Variable	Category or (unit)	mQC method (n=13)	DP method (n=8)
Median age (IQR)	Years	73.0 (42.0, 89.0)	80.0 (50.0, 90.0)
Etiology n (%)	Exogenous	3 (23.1)	1 (12.5)
	Infectious	5 (38.5)	3 (37.5)
	Cardiac arrest	1 (7.7)	2 (25.0)
	Others	4 (30.8)	2 (25.0)
Sex n (%)	Male	9 (69.2)	5 (62.5)
	Female	4 (30.8)	3 (37.5)
Weight median (IQR)	kg	50.00 (38.00, 80.00)	53.50 (38.00, 70.00)
Sedative use n (%)	No	3 (23.1)	7 (87.5)
	Yes	10 (76.9)	1 (12.5)
Analgesic use n (%)	No	10 (76.9)	5 (62.5)
	Yes	3 (23.1)	3 (37.5)
Administration route n (%)	Peripheral	12 (80.0)	6 (75.0)
	Central	3 (23.1)	2 (25.0)
Diastolic blood pressure median (IQR)	mm Hg	65.00 (41.00, 76.00)	58.00 (42.00, 89.00)
Mean arterial pressure median (IQR)	mm Hg	77.00 (58.00, 96.00)	73.50 (49.00, 107.00)
Systolic blood pressure median (IQR)	mm Hg	109.00 (79.00, 133.00)	92.50 (67.00, 125.00)
Norepinephrine infusion rate median (IQR)	mg/mL/hour	0.23 (0.06, 0.57)	0.25 (0.16, 0.40)

Outcome

**Figure 3:**
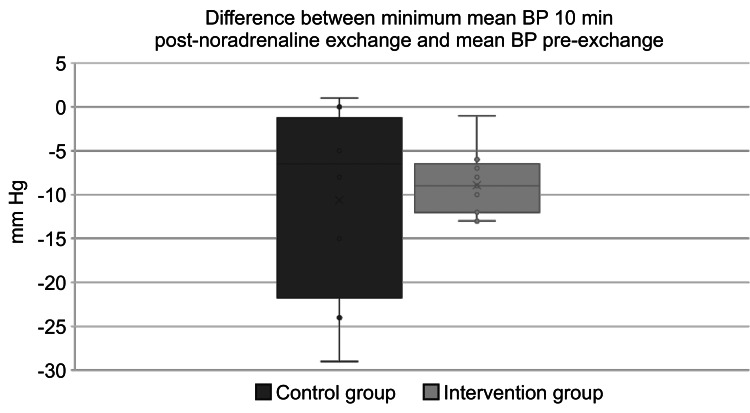
Difference between minimum mean BP 10 minutes post-noradrenaline exchange and mean BP pre-exchange BP: blood pressure; min: minutes

**Figure 4:**
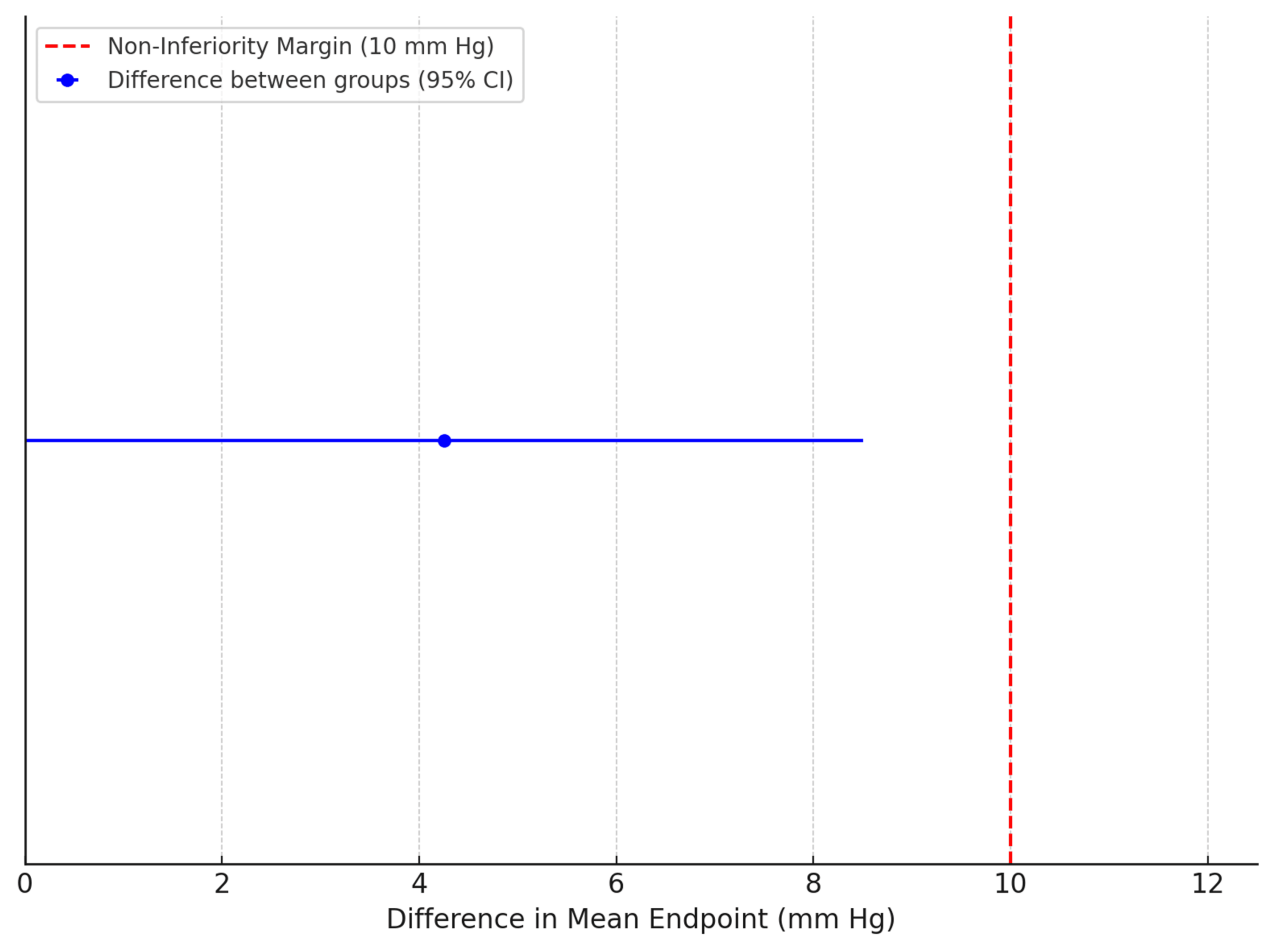
Non-inferiority assessment of the mQc method compared to the DP method: difference in mean endpoint with 95% CI mQc: modified quick change method; DP: double pumping method; 95%CI: 95% confidence interval

The mean value of the primary endpoint was −8.92 mm Hg in the mQC group and −10.63 mm Hg in the DP group (Figure 3). The 95% confidence interval for the absolute difference ∣P1−P0∣ was 0 mmHg to 8.50 mmHg. As the upper limit of the 95% confidence interval did not exceed the prespecified non-inferiority margin of 10 mmHg, the mQC method was verified to be non-inferior compared to the DP method (Figure 4).

## Discussion

This quasi-randomized trial verified that the mQC method, which combines the QC method with a 4 mL discard technique, was non-inferior to the conventional DP method in patients receiving continuous intravenous norepinephrine infusion. Considering its safety and relative simplicity, the mQC method has the potential to become a standard exchange technique for continuous intravenous norepinephrine administration.

Several studies, including prospective comparative ones, have been conducted to examine norepinephrine syringe exchange [18-20]. Barbieri et al. compared the DP and QC methods in a randomized controlled trial that showed no significant difference in blood pressure fluctuations between them; they recommended using the simpler QC method [14]. A larger study demonstrated that the QC method suppressed blood pressure fluctuations [15]. Based on this evidence, using the QC method appears most rational. However, in Japan, the DP method is nevertheless frequently implemented simply based on custom [13].

The key factors in minimizing hemodynamic effects during norepinephrine exchange are (1) minimizing the length of time that the intravenous infusion is interrupted during the exchange and (2) stabilizing the drug concentration in the circuit by maintaining the flow rate. The DP method theoretically has no interruption time because norepinephrine is being administered simultaneously via two parallel routes. Interruption may occur with the QC method; however, when switching the administration route, it may not. Nevertheless, the open three-way stopcocks commonly used in Japan are expected to minimize concentration changes during the exchange. Additionally, the sliding resistance in new syringes can affect the norepinephrine flow rate [16,21,22]. To address this, the mQC method combines the QC method with a technique of discarding 4 mL from the newly filled norepinephrine syringe. Although Yoshimura et al. conducted a comparative study using this method, it involved only four cases and used glucose rather than norepinephrine [17]. The novelty of our study lies in the use of norepinephrine and examining safety.

The mQC method is simpler than the DP method, which is important, considering that medication administration-related errors account for a large proportion of adverse events [23], and errors involving vasoactive drugs such as norepinephrine can have serious hemodynamic consequences. Therefore, a simpler technique with ensured safety is crucial for the prevention of adverse events.

Another strength of this study was the administration of a constant flow (10 mL/hour) of fluid through the same route as the norepinephrine infusion in both groups, which prevented delayed drug delivery caused by the startup curve effect [24]. This ensured that differences in syringe pump characteristics did not influence the results of either group.

Our study had several limitations. First, it was quasi-randomized in design, allocating the intervention based on time period. Unlike standard randomization, quasi-randomization lacks unpredictability and may not ensure an equal distribution of unknown confounders, potentially reducing internal validity. Second, the DP group’s sample size did not reach the predetermined number of 13, which may have affected the precision of the treatment effect estimate by widening the confidence interval. Third, blinding to the method of syringe exchange was simply not possible owing to its nature. Finally, the two groups were heterogeneous with respect to various conditions (infections, trauma, etc.), which may have differentially influenced circulatory dynamics. However, sample size constraints precluded statistically valid sensitivity analysis.

## Conclusions

The mQC method of norepinephrine syringe exchange in critically ill patients was not inferior to the DP method in terms of its effect on hemodynamic stability. However, confounding bias may have been present considering the small sample size and the study’s non-blinded, quasi-randomized design. Further verification in large-scale randomized controlled trials is necessary.
